# Impact of Firearm Surveillance on Gun Control Policy: Regression Discontinuity Analysis

**DOI:** 10.2196/26042

**Published:** 2021-04-22

**Authors:** Lori Post, Maryann Mason, Lauren Nadya Singh, Nicholas P Wleklinski, Charles B Moss, Hassan Mohammad, Tariq Z Issa, Adesuwa I Akhetuamhen, Cynthia A Brandt, Sarah B Welch, James Francis Oehmke

**Affiliations:** 1 Buehler Center for Health Policy and Economics Feinberg School of Medicine Northwestern University Chicago, IL United States; 2 Feinberg School of Medicine Northwestern University Chicago, IL United States; 3 Institute of Food and Agricultural Sciences University of Florida Gainsville, FL United States; 4 Yale Center for Medical Informatics Yale School of Medicine Yale University New Haven, CT United States

**Keywords:** firearm surveillance, assault weapons ban, large-capacity magazines, guns control policy, mass shootings, regression lines of discontinuity

## Abstract

**Background:**

Public mass shootings are a significant public health problem that require ongoing systematic surveillance to test and inform policies that combat gun injuries. Although there is widespread agreement that something needs to be done to stop public mass shootings, opinions on exactly which policies that entails vary, such as the prohibition of assault weapons and large-capacity magazines.

**Objective:**

The aim of this study was to determine if the Federal Assault Weapons Ban (FAWB) (1994-2004) reduced the number of public mass shootings while it was in place.

**Methods:**

We extracted public mass shooting surveillance data from the Violence Project that matched our inclusion criteria of 4 or more fatalities in a public space during a single event. We performed regression discontinuity analysis, taking advantage of the imposition of the FAWB, which included a prohibition on large-capacity magazines in addition to assault weapons. We estimated a regression model of the 5-year moving average number of public mass shootings per year for the period of 1966 to 2019 controlling for population growth and homicides in general, introduced regression discontinuities in the intercept and a time trend for years coincident with the federal legislation (ie, 1994-2004), and also allowed for a differential effect of the homicide rate during this period. We introduced a second set of trend and intercept discontinuities for post-FAWB years to capture the effects of termination of the policy. We used the regression results to predict what would have happened from 1995 to 2019 had there been no FAWB and also to project what would have happened from 2005 onward had it remained in place.

**Results:**

The FAWB resulted in a significant decrease in public mass shootings, number of gun deaths, and number of gun injuries. We estimate that the FAWB prevented 11 public mass shootings during the decade the ban was in place. A continuation of the FAWB would have prevented 30 public mass shootings that killed 339 people and injured an additional 1139 people.

**Conclusions:**

This study demonstrates the utility of public health surveillance on gun violence. Surveillance informs policy on whether a ban on assault weapons and large-capacity magazines reduces public mass shootings. As society searches for effective policies to prevent the next mass shooting, we must consider the overwhelming evidence that bans on assault weapons and/or large-capacity magazines work.

## Introduction

### Background

Approximately 44,000 people are killed and an additional 100,000 people are injured by a gun each year in the United States [1,2]. Mass shooting fatalities, as a particular type of gun injury event, account for <1% of all gun deaths [3] and have largely been ignored until recently [4,5]; yet, mass shooting events occur multiple times per year [6]. This information is based on insights from firearm surveillance performed by a variety of researchers, and state and federal agencies on incidence, prevalence, risk factors, injuries, deaths, and precipitating events, similar to the surveillance of infectious diseases such as COVID-19 [7-21]. Teutch and Thacker [22] defined public health surveillance as

the ongoing systematic collection, analysis, and interpretation of health data, essential to the planning, implementation, and evaluation of public health practice, closely integrated to the dissemination of these data to those who need to know and linked to prevention and control.

Not only do surveillance systems generate hypotheses to test but they also provide the data to test them.

The Federal Assault Weapons Ban (FAWB, also known as the Public Safety and Recreational Firearms Use Protection Act) included a ban on the manufacture for civilian use or sale of certain semiautomatic firearms defined as assault weapons as well as certain large-capacity magazines (LCMs). The Act was in effect for 10 years from 1994 until it sunsetted in 2004. Semiautomatic weapons (rapid fire) and assault weapons (second grip plus other features) are distinct; however, the two are often incorrectly conflated as similar [23-26]. Semiautomatic weapons are defined as weapons that automatically load another cartridge into a chamber, preparing the weapon for firing, but requiring the shooter to manually release and press the trigger for each round [23-26]. By contrast, automatic weapons are similarly self-loading, but allow for a shooter to hold the trigger for continuous fire [27]. Furthermore, the FAWB also prohibited certain ammunition magazines that were defined as “large-capacity” cartridges [28] containing more than 10 bullets [29]. These LCMs can feed ammunition to semiautomatic weapons that do not meet the criteria of being considered assault weapons. Furthermore, LCMs are considered one of the most important features of the FAWB as research has found a relationship between bans on LCMs and casualty counts at the state level [30-34]. The 10-year federal ban was signed into law by President Clinton on September 13, 1994 [28].

Firearm surveillance data have been used to test potential policy responses to prevent mass shootings, including the FAWB [32,34-39], Extreme Risk Protection Orders (also known as red flag laws) [40-45], and federal and state LCM bans [31,32,46]. In particular, it seems likely that the FAWB and LCM bans have potential to affect mass shootings because they regulate weapons and ammunition formats that are designed to enable rapid discharge, which is a key feature in mass shooting incidents [24,47]. Other types of gun deaths may not be responsive to the FAWB or LCM bans. As an example, Extreme Risk Protection Orders or “Red Flag” orders [43,48], which temporarily prohibit at-risk individuals from owning or purchasing firearms, may be effective for preventing firearm suicides or domestic violence homicides [49] but less effective for public mass shooters [50,51]. The prohibition of LCMs may have no impact on firearm suicide because suicide decedents only require one bullet to kill themselves [52].

Several studies during and after the FAWB attempted to determine if gun policy that restricts the production and sale of assault weapons and LCMs decreased gun deaths [53,54]. These initial studies make meaningful contributions to the literature because they describe what constitutes assault weapons, magazine capacity, ballistics, and loopholes in the FAWB legislation [3,53-57]. However, these studies have found little to no evidence that these policies have had any overall effect on firearm homicides, gun lethality, or overall crime [58-61]. Since deaths from public mass shootings comprise less than 1% of all homicides based on our definition, testing whether or not the FAWB/LCM ban has an impact on homicide would wash out the effect. Since the FAWB/LCM ban may be effective at specific types of gun deaths, sampling must be limited to specific types of shooters over overall gun deaths or tests for lethality [62,63]. Finally, the variation in research findings is related to differences in research design, sampling frame, and case definition of a public mass shooting [3,53-56,64,65].

Our study differs from other studies that evaluated the efficacy of the FAWB because we used economic methods and a different outcome variable. Specifically, we focused on whether the FAWB resulted in fewer public mass shooting “events,” whereas other studies evaluated the number of gun injuries and deaths that occurred during the course of a mass shooting.

### Objective

The aim of this study was to test whether curbing *access to certain types of guns and magazines* will decrease mass shooting *events*. We sought to empirically answer if there was a relationship between the FAWB and a reduction in mass shooting events.

## Methods

### Data Source

We created a firearm surveillance system based on the National Institute of Justice–funded Violence Project dataset, which culled mass shooting events from 1966 to 2019 [6]. Consistent with earlier studies, we rely on the original Federal Bureau of Investigation (FBI) definition of a massacre, specifically where 4 or more people are killed within a single timeframe. We differentiate our mass shootings from others in that our inclusion criteria require the shootings to have occurred in a public setting. We adapted this definition to only include massacres that involved gun deaths of 4 or more victims to isolate a particular type of mass shooter [66]. Many firearm surveillance systems that include mass shootings use a lower threshold of persons shot and many do not include deaths. An FBI report on active shooters in mass shooting events identified planning and preparation behaviors that are central to prevention [67]. This more narrow definition isolates premeditation, whereas broader definitions may include shooters that are more reactive [68]. Our case definition does not include family annihilators or felony killers because *familicides are defined by the victim-offender relationship, public massacres are defined by location, and felony killings are distinguished by motive* [69]. This differentiation is consistent with other mass shooting studies [70-72].

We examined the annual number of public mass shootings occurring between 1966 and 2019 that resulted in 4 or more fatalities. The hypothesis was that the FAWB reduced the number of public mass shootings per year during the period of the ban. We used regression discontinuity analysis to test the hypothesis. Regression discontinuity analysis is a standard economist tool used in policy analysis taking advantage of quasi-experimental designs [65,73].

### Analyses

Regression discontinuity analysis allows for discontinuities or shifts in both the intercept and the slope of the trend line at both the onset and sunset of the FAWB. That is, we introduced intercept shift parameters in 1995 and 2005, and trend shift parameters for the periods 1995-2004 and 2005-2019. A statistically significant shift in a parameter indicates a discontinuity (ie, a finding that the FAWB had a statistically significant effect on the number of public mass shootings). We tested for statistical significance of the intercept and trend shift parameters both independently and jointly. All statistical inference was based on a significance level set at .05. We used the Huber-White robust residuals, which attenuate problems of autocorrelation, heteroscedasticity, and some types of model misspecification [74].

We then used the estimated model for two types of counterfactual analysis. First, we used the model to predict the number of public mass shootings that would have occurred had the FAWB not been in place. The difference between this counterfactual prediction and the modeled number of incidents with the FAWB in place provided an estimate of the number of public mass shootings that the FAWB prevented.

Second, we projected forward the number of public mass shootings that would have occurred had the FAWB been permanent (ie, continued from 2004 through to the end of the sample period). We note that in some sense, this is an “out of sample” exercise because even though the sample extends to 2019, the FAWB ended in 2004; thus, this exercise would not pick up events in the past 15 years that would have augmented or compromised the effects of the FAWB. The difference between the modeled number of public mass shootings and the projected counterfactual number of public mass shootings could provide an estimate of the number of public mass shootings that the FAWB prevented.

We performed a regression of the 5-year moving average of public mass shootings on the US population in millions, the homicide rate, and discontinuity variables to capture both the effects of the FAWB and its discontinuation. We did not introduce a trend line for the entire sample period because it is highly collinear with the population variable. For the period of the FAWB’s implementation, we originally introduced an intercept shift, time trend, and shift in the homicide rate; for the post-FAWB period, we introduced an intercept shift and a time trend. Due to collinearity, we retained only the trend shift in the final model for the FAWB period; for the post-FAWB period, we retained both the intercept and the trend shift.

## Results

**Figure 1:**
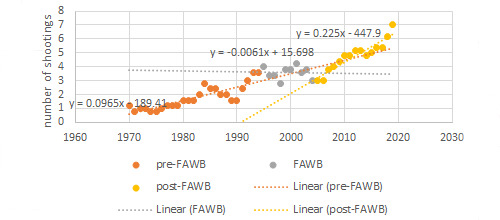
Public mass shooting trend line using five year moving averages (1966-2019).

We identified a total of 170 public mass shooting events, the primary outcome variable, with 4 or more fatalities between 1966 and 2019. The 5-year cumulative number of public mass shootings is shown in Figure 1, providing a visualization of the impacts of the FAWB on the number of shootings. The first mass shooting occurred in 1966; hence, the first data point for the cumulative number of shootings over the previous 5 years occurs in 1970. For 1966 and 1967, the cumulative number of public mass shootings was 3. This number then increased to 12 in 1993 and declined to 3 in 2004. After 2004, the cumulative number of public mass shootings increased to 81 in 2019. The last year of the ban, 2004, experienced the fewest public mass shootings through 2019.

The regression results showed excellent explanatory power (R^2^=0.94). The coefficient on population was positive and statistically significant (.044, *P*<.001). This coefficient means that for every increase in population of 1 million people, there are an additional .044 public mass shooting events per year. The coefficient on the homicide rate was negative and statistically significant (–.249, *P*=.01). The coefficient on the time trend for the FAWB period captures the effect of the FAWB; this coefficient was negative and statistically significant (–.187, *P*=.001). Using prediction models in combination with regression slopes, we estimate that 11 public mass shootings were avoided due to the FAWB. The intercept discontinuity for 2005-2019 was negative and statistically significant (–2.232, *P*=.001), and the trend coefficient was positive and statistically significant (.081, *P*=.001).

**Figure 2:**
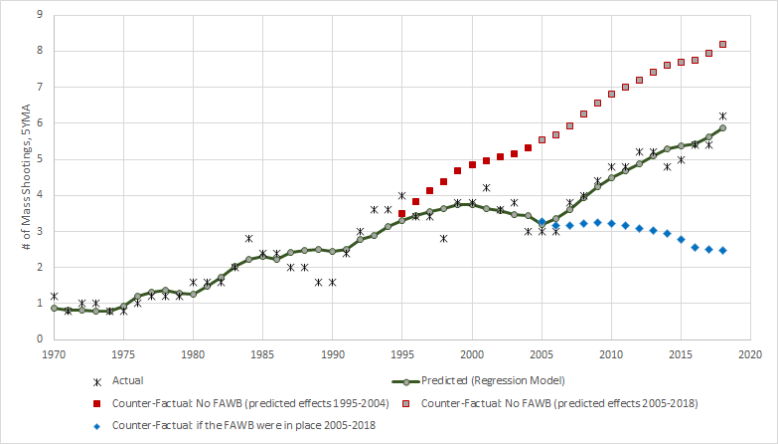
Regression lines from discontinuity analysis of the federal assault weapons ban (1994-2004).

These results are graphed in Figure 2 in which the black stars represent the actual data and the green line represents the predicted numbers of public mass shootings from the regression discontinuity model. A bending of the trend during the FAWB period to become downward sloping at the end of the period is apparent, as is the return of the upward trajectory upon expiration of the FAWB. The red squares represent the projected numbers of public mass shootings during the FAWB period had there been no FAWB. The difference between the red squares and the green lines represents the predicted number of public mass shootings averted by the FAWB. The model predicts that 11 public mass shootings were averted over the period of 1995-2004.

The blue diamonds represent the projected effects of a continuation of the FAWB through 2019 based on the observed trend from 1995 to 2004. This projection indicates that 30 public mass shootings would have been prevented from 2005 to 2019 had the FAWB been left in place.

## Discussion

### Principal Findings

In total, 1225 people were killed in a mass shooting over the past 53 years with more than half occurring in the last decade, a function of increases in mass shootings and weapon lethality [62,63,75]. Public mass shooting fatalities and injuries far outpace population growth [75]. Between 1966 and 2019, the US population increased by 67% [76], whereas public mass shooting deaths increased by over 5-fold. The rise in public mass shootings throughout the sample period is in fact partially a function of population growth and homicide rate, along with the effects of the FAWB and its removal. An increase in the US population of 1 million people was associated with an increase of .040 (*P*<.005) public mass shootings per year. During the post-FAWB period, the increase in population from approximately 300 million in 2005 to 330 million in 2019 should be associated with an increase of 1.2 public mass shootings per year, compared to the actual increase of 4 public mass shootings per year in the data (5-year moving average). After controlling for population growth and homicide rate, a positive and statistically significant coefficient (.081, *P*=.001) on the 2005-2018 trend was seen. This further indicates a separate, nonpopulation trend of increasing violence operating during the post-FAWB period. The negative coefficient on the homicide rate invalidates the hypothesis that decreases in the numbers of public mass shootings are simply reflections of an overall decreasing homicide rate. The negative intercept discontinuity is consistent with an effect of the FAWB that persists somewhat beyond the immediate end of the ban. The positive trend coefficient is consistent with the hypothesis that the FAWB was associated with a decrease in the number of public mass shootings, as the expiration of the FAWB was associated with a shift from a downward trend to an upward trend in the number of public mass shootings per year.

The most striking finding from this study is that there was a reduction in the number of public mass shooting events while the FAWB was in place. Using prediction models in combination with regression slopes, we estimate that 11 public mass shootings were avoided due to the FAWB. By projecting what would have happened if the FAWB remained in place, we found that there would have been significantly fewer public mass shootings if the FAWB had remained in place to 2019. Remarkably, although it is intuitive that the removal of assault weapons and magazine clips will reduce the lethality of a mass shooting, we observed an inverse relationship between weapons/ammunition and mass shooting events, meaning that mass shooters may be less likely to perpetrate a mass shooting without rapid fire military-style weapons. This is an independent effect, which indirectly leads to fewer injuries and deaths. DiMaggio et al [64] also found evidence of a decrease in public mass shootings during the ban; however, their study period was shorter and was restricted to 51 public mass shootings. Unlike our study, they implicitly modeled public mass shootings as a random instance of general gun homicides that had a high death count [64]. In contrast, our findings suggest that public mass shootings are a unique type of premeditated gun violence. We found that prior to enactment of the FAWB, the rate of public mass shootings was increasing. During enactment of the FAWB, there was a downward trend of mass shooting events. After the FAWB was lifted, public mass shootings increased dramatically. Firearm homicides in general follow no such patterns.

This effect was not found in the work of Koper, Roth, and colleagues [53-55]; however, their inclusion of all gun homicides masks the ban’s effect on mass shootings. Even though Peterson and Densley’s [77] work focused on perpetrator histories and not the FAWB, their findings that ease of gun access is characteristic of public mass shooters further supports our study. We restricted the inclusion criteria to public mass shootings to specifically test the effectiveness of the FAWB on public mass shooting events.

Regardless of the FAWB, bringing a semiautomatic rifle with high magazine capacity to a massacre significantly increases the number of fatalities and injuries. The increase in deaths is a function of rapid fire and increased ballistic energy. The increase in injuries is also a function of rapid fire and high-capacity magazines, enabling the shooter to shoot more people in crowded venues quickly before the crowd can disperse or hide. When controlling for the FAWB, the use of assault rifles decreased by half during implementation of the ban and tripled after the ban was lifted. This is a particularly important finding given that the FAWB had loopholes and that overall violent crime is decreasing [78]. First, all people with an assault weapon prior to the FAWB were allowed to retain their semiautomatic weapons [54,64]. Second, without a buyback program, semiautomatic weapons remained in the community [54,64]. Third, the ban did not target some military assault-like weapons [54,64]. Finally, a major loophole found in gun control legislation is that buyers can bypass background checks by purchasing their weapons and ammunition from gun shows, through illegal purchasing, or legally purchasing their guns and ammunition from another gun owner [57,63,79-87]. Even with these loopholes and issues, there was still a significant reduction in public mass shootings during the FAWB. These loopholes indicate that most people who purchase assault weapons do not become mass shooters; however, mass shooters require assault weapons and LCMs to carry out a mass shooting. Ban effectiveness might have improved if all assault weapons were included in the FAWB.

Some recent studies have specifically analyzed the effects of LCM bans on the incidence of public mass shootings. In a review of state legislation, Webster et al [88] found that bans of LCMs were associated with a significant reduction in the incidence of fatal public mass shootings. This study shows that the FAWB, which included a ban on LCMs, was associated with fewer fatalities and injuries during mass shootings in addition to fewer public mass shooting events. Koper et al [27] previously reported that 19% of public mass shootings resulting in 4 or more fatalities included the use of LCMs, while only 10% involved an assault weapon. Klarevas et al [29] found a similar pattern in shootings of 6 or more people, in which 67% of shooters utilized LCMs, whereas only 26% utilized an assault weapon. Because our study only looked at effects of the FAWB, which included an LCM ban, we were only able to determine the combined effects of limiting assault weapons and LCMs. To be clear, the reduction in the number of public mass shootings, and resulting fatalities and injuries, may be a function of the ban on assault weapons, assault weapons plus LCMs, or only LCMs. We cannot separate out their independent effects at the national level.

Unlike our study, Webster et al [88] did not evaluate the incidence of assault weapons used in public mass shootings. Rather, they focused on fatalities from public mass shootings vs public mass shooting events. Although Webster et al [88] utilized the FBI Supplemental Homicide Report as their dataset, which is a voluntary reporting measurement system prone to errors in reporting, their findings are applicable to our analysis.

### Limitations

Although we found statistically significant decreases during the FAWB, we cannot isolate aspects of the policy that are attributed to the decline. Most notably, the FAWB also included LCMs during the ban. It may be that the type of gun and/or the type of magazine resulted in a decline. Indeed, assault weapons and LCMs provide the means to carry out a mass shooting; however, there are likely other factors beyond this study that partially explain the radical increase in public mass shootings in the post-FAWB period. For example, the FAWB was in place from 1994 to 2004, which is the same time period that the US population largely adopted the internet, along with associated social communication software and websites. This may have resulted in better tracking of public mass shootings or increased media coverage. Because our study specifically targeted the federal legislation, we omitted state-level gun policies such as state-level prohibitions on certain types of guns, LCMs, or more lethal types of bullets. It is likely that the internet serves as a contagion and as a guide to potential mass shooters, allowing them to access weapons and multiple stories about other mass shooters [62,67,89,90].

### Conclusions

In summary, public mass shootings are a unique and specific type of homicide by a gun. We found evidence that public mass shootings are qualitatively different from general homicides because after the FAWB expired, mass shooting events increased while general homicides decreased. The increase in public mass shootings was more dramatic in the final 10 years of the study period following the end of the FAWB. We suspect that these outcomes may be improved by removing existing semiautomatic weapons with large bullet capacity by creating a buyback program for all rapid-firing weapons. Moreover, the legislation would be strengthened if it closed loopholes that allow gun buyers to get around the background check legislation and other purchase prohibitions by exempting gun shows and internet or person-to-person purchases, which were exempted from the FAWB and LCM ban [87].

## Abbreviations

FAWB: Federal Assault Weapons Ban
FBI: Federal Bureau of Investigation
LCM: large-capacity magazine

## References

[1] (2020). Web-based Injury Statistics Query and Reporting System. Centers for Disease Control and Prevention, Injury Prevention and Control.

[2] Christensen AJ, Cunningham R, Delamater A, Hamilton N (2019). Introduction to the special issue on gun violence: addressing a critical public health challenge. J Behav Med.

[3] Drake B (2013). Mass shootings rivet national attention, but are a small share of gun violence. Pew Research Center.

[4] Bowers TG, Holmes ES, Rhom A (2009). The nature of mass murder and autogenic massacre. J Police Crim Psych.

[5] Delisi M, Scherer AM (2016). Multiple homicide offenders. Crim Justice Behav.

[6] (2020). Mass shooter database. The Violence Project.

[7] Shelby D (2021). Association between adult alcohol misuse, adult mental health, and firearm storage practices in households with children: findings from the Behavioral Risk Factor Surveillance System (BRFSS). MPH Thesis. ScholarWorks @ Georgia State University.

[8] Loder R, Mishra A, Atoa B, Young A (2021). Spinal injury associated with firearm use. Cureus.

[9] Mueller KL, Trolard A, Moran V, Landman JM, Foraker R (2021). Positioning public health surveillance for observational studies and clinical trials: The St. Louis region-wide hospital-based violence intervention program data repository. Contemp Clin Trials Commun.

[10] Horn DL, Butler EK, Stahl JL, Rowhani-Rahbar A, Littman AJ (2021). A multi-state evaluation of the association between mental health and firearm storage practices. Prev Med.

[11] Gunn JF, Boxer P (2021). Gun laws and youth gun carrying: results from the youth risk behavior surveillance system, 2005-2017. J Youth Adolesc.

[12] Rozel J, Soliman L, Jain A, Zun LS, Nordstrom K, Wilson MP (2021). The gun talk: how to have effective conversations with patients and families about firearm injury prevention. Behavioral Emergencies for Healthcare Providers.

[13] Keyes KM, Kandula S, Olfson M, Gould MS, Martínez-Alés G, Rutherford C, Shaman J (2021). Suicide and the agent–host–environment triad: leveraging surveillance sources to inform prevention. Psychol Med.

[14] Bluestein G, Hallerman T, Lee LK, Fleeger EW (2021). Future directions for firearm injury intervention, policy, and research. Pediatric Firearm Injuries and Fatalities: The Clinician's Guide to Policies and Approaches to Firearm Harm Prevention.

[15] Oehmke J, Moss C, Singh L, Oehmke T, Post L (2020). Dynamic panel surveillance of COVID-19 transmission in the United States to inform health policy: observational statistical study. J Med Internet Res.

[16] Oehmke J, Oehmke T, Singh L, Post L (2020). Dynamic panel estimate-based health surveillance of SARS-CoV-2 infection rates to inform public health policy: model development and validation. J Med Internet Res.

[17] Post L, Benishay E, Moss C, Murphy R, Achenbach C, Ison M, Resnick D, Singh LN, White J, Chaudhury AS, Boctor MJ, Welch SB, Oehmke JF (2021). Surveillance metrics of SARS-CoV-2 transmission in central Asia: longitudinal trend analysis. J Med Internet Res.

[18] Post LA, Issa TZ, Boctor MJ, Moss CB, Murphy RL, Ison MG, Achenbach CJ, Resnick D, Singh LN, White J, Faber JMM, Culler K, Brandt CA, Oehmke JF (2020). Dynamic public health surveillance to track and mitigate the US COVID-19 epidemic: longitudinal trend analysis study. J Med Internet Res.

[19] Post LA, Lin JS, Moss CB, Murphy RL, Ison MG, Achenbach CJ, Resnick D, Singh LN, White J, Boctor MJ, Welch SB, Oehmke JF (2021). SARS-CoV-2 wave two surveillance in East Asia and the Pacific: longitudinal trend analysis. J Med Internet Res.

[20] Post L, Marogi E, Moss CB, Murphy RL, Ison MG, Achenbach CJ, Resnick D, Singh L, White J, Boctor MJ, Welch SB, Oehmke JF (2021). SARS-CoV-2 surveillance in the Middle East and North Africa: longitudinal trend analysis. J Med Internet Res.

[21] Post LA, Argaw ST, Jones C, Moss CB, Resnick D, Singh LN, Murphy RL, Achenbach CJ, White J, Issa TZ, Boctor MJ, Oehmke JF (2020). A SARS-CoV-2 surveillance system in Sub-Saharan Africa: modeling study for persistence and transmission to inform policy. J Med Internet Res.

[22] Teutsch S, Thacker S (1995). Planning a public health surveillance system. Epidemiol Bull.

[23] Jacobs J, Fuhr Z (2017). The Safe Act: New York's ban on assault weapons and large capacity magazines. Crim Law Bull.

[24] Wallace EG (2018). Assault weapon myths. South Ill Univ Law J.

[25] Kopel D, Lowy J, Rostron A (2018). Heller and "Assault Weapons". Campbell Law Rev.

[26] Pfau MW (2020). Defining the deadly: definitional argument and the assault weapons ban controversy. Argum Advocacy.

[27] Koper CS, Johnson WD, Nichols JL, Ayers A, Mullins N (2018). Criminal use of assault weapons and high-capacity semiautomatic firearms: an updated examination of local and national sources. J Urban Health.

[28] United States Congress House Committee on the Judiciary. Subcommittee on Crime and Criminal Justice (1994). Public Safety and Recreational Firearms Use Protection Act. Hearing before the Subcommittee on Crime and Criminal Justice of the Committee on the Judiciary, House of Representatives, One Hundred Third Congress, second session, on H.R. 3527.

[29] Klarevas L, Conner A, Hemenway D (2019). The effect of large-capacity magazine bans on high-fatality mass shootings, 1990–2017. Am J Public Health.

[30] Kleck G (2016). Large-capacity magazines and the casualty counts in mass shootings. Justice Res Policy.

[31] Abbasi J (2020). Large-capacity magazine bans linked with fewer mass shootings, deaths. JAMA.

[32] Koper CS (2020). Assessing the potential to reduce deaths and injuries from mass shootings through restrictions on assault weapons and other high‐capacity semiautomatic firearms. Criminol Public Policy.

[33] Towers S, Wallace D, Hemenway D (2019). Temporal trends in public mass shootings: high-capacity magazines significantly increase fatality counts, and are becoming more prevalent. medRxiv preprint server.

[34] Webster DW, McCourt AD, Crifasi CK, Booty MD, Stuart EA (2020). Evidence concerning the regulation of firearms design, sale, and carrying on fatal mass shootings in the United States. Criminol Public Policy.

[35] Lowy J (2020). Comments on assault weapons, the right to arms, and the right to live. Harv J Law Public Policy.

[36] Kim A (2020). United States gun policy and the effect on mass shootings. California State University Northridge Scholarworks Open Access Repository.

[37] Pfau MW (2020). Defining the deadly: definitional argument and the assault weapons ban controversy. Argum Advocacy.

[38] Balakrishna M, Wilbur KC (2021). How the Massachusetts Assault Weapons Ban Enforcement Notice changed firearm sales. SSRN J.

[39] Soto L, Chheda S, Soto J (2020). Reducing fatalities in mass attacks and the related matter of gun control policy following the El Paso August 2019 shooting. Tex Hisp J Law Policy.

[40] Nagin DS, Koper CS, Lum C (2020). Policy recommendations for countering mass shootings in the United States. Criminol Public Policy.

[41] Gay C (2020). 'Red Flag' laws: how law enforcement's controversial new tool to reduce mass shootings fits within current Second Amendment jurisprudence. Boston Coll Law Rev.

[42] Nielsen D (2020). Disarming dangerous persons: how Connecticut's Red Flag Law saves lives without jeopardizing constitutional protections. Quinnipiac Health Law J.

[43] Blocher J, Charles J (2020). Firearms, extreme risk, and legal design: “Red Flag” laws and due process. Virginia Law Rev.

[44] Kopel DB (2020). Red Flag Laws: proceed with caution. Law Psychol Rev.

[45] Blodgett S (2020). Dementia, guns, Red Flag laws: Can Indiana's Statute balance elders' constitutional rights and public safety?. NAELA J.

[46] Kerr S, Crews G (2019). "What We Need Is Bullet Control": could regulation of bullets reduce mass shootings?. Handbook of Research on Mass Shootings and Multiple Victim Violence.

[47] Moore EE (2018). Another mass shooting: Time to ban the assault rifle. J Trauma Acute Care Surg.

[48] Delaney GA, Charles JD (2020). A double-filter provision for expanded Red Flag laws: a proposal for balancing rights and risks in preventing gun violence. J Law Med Ethics.

[49] Honberg RS (2020). Mental illness and gun violence: research and policy options. J Law Med Ethics.

[50] Laqueur HS, Wintemute GJ (2019). Identifying high‐risk firearm owners to prevent mass violence. Criminol Public Policy.

[51] Pallin R, Schleimer JP, Pear VA, Wintemute GJ (2020). Assessment of extreme risk protection order use in California from 2016 to 2019. JAMA Netw Open.

[52] Hurka S, Knill C (2018). Does regulation matter? A cross‐national analysis of the impact of gun policies on homicide and suicide rates. Regul Gov.

[53] Koper C, Roth J (2001). The impact of the 1994 Federal Assault Weapon Ban on gun violence outcomes: an assessment of multiple outcome measures and some lessons for policy evaluation. J Quant Criminol.

[54] Koper C, Woods D, Roth J (2004). Updated Assessment of the Federal Assault Weapons Ban: Impacts on Gun Markets and Gun Violence, 1994-2003. Report to the National Institute of Justice, United States Department of Justice.

[55] Roth J, Koper C, Adams W, Johnson S, Marcotte J, McGready J, Scott A, Valera M, Wissoker D (1997). Impact Evaluation of the Public Safety and Recreational Firearms Use Protection Act of 1994 Final Report. Urban Institute.

[56] Webster D, Vernick J, McGinty E, Alcorn T (2013). Regulating Gun Sales: An Excerpt from Reducing Gun Violence in America: Informing Policy with Evidence and Analysis.

[57] Jacobs J (2004). Can Gun Control Work? (Studies in Crime and Public Policy).

[58] Lee LK, Fleegler EW, Farrell C, Avakame E, Srinivasan S, Hemenway D, Monuteaux MC (2017). Firearm laws and firearm homicides: a systematic review. JAMA Intern Med.

[59] Gius M (2013). An examination of the effects of concealed weapons laws and assault weapons bans on state-level murder rates. Appl Econ Lett.

[60] Cook P, Goss K (2014). The Gun Debate: What Everyone Needs to Know.

[61] Cook P, Goss K (2020). The Gun Debate: What Everyone Needs to Know, 2nd Edition.

[62] Lankford A, Silver J (2019). Why have public mass shootings become more deadly?. Criminol Public Policy.

[63] Schiff M (2019). IZA Discussion Paper 12784: Greater US gun ownership, lethality and murder rates: analysis and policy proposals. IZA Institute of Labor Economics.

[64] DiMaggio C, Avraham J, Berry C, Bukur M, Feldman J, Klein M, Shah N, Tandon M, Frangos S (2019). Changes in US mass shooting deaths associated with the 1994–2004 federal assault weapons ban: Analysis of open-source data. J Trauma Acute Care Surg.

[65] (2021). World Telecommunication/ICT Indicators Database 2020 (24th Edition). ​​​​​​​​​​​​​​​​​​​​​International Telecommunications Union.

[66] Lopez G (2021). America's unique gun violence problem, explained in 17 maps and charts. Vox.

[67] Silver J, Simons A, Craun S (2018). A study of the pre-attack behaviors of active shooters in the United States between 2000 and 2013. FBI Documents.

[68] DeFoster R, Swalve N (2018). Guns, culture or mental health? Framing mass shootings as a public health crisis. Health Commun.

[69] Fridel EE (2021). A multivariate comparison of family, felony, and public mass murders in the United States. J Interpers Violence.

[70] Duwe G (2007). Mass murder in the United States: a history.

[71] Fox J, Levin J (2015). Mass confusion concerning mass murder. Criminologist.

[72] Fox JA, Levin J (2016). Firing back: the growing threat of workplace homicide. An Am Acad Pol Soc Sci.

[73] Stevenson AJ, Flores-Vazquez IM, Allgeyer RL, Schenkkan P, Potter JE (2016). Effect of removal of Planned Parenthood from the Texas Women’s Health Program. N Engl J Med.

[74] Freedman DA (2006). On the so-called “Huber Sandwich Estimator” and “Robust Standard Errors”. Am Statistician.

[75] Duwe G (2017). Mass shootings are getting deadlier, not more frequent. Politico Magazine.

[76] (2019). Population Trends. United States Census Bureau.

[77] Peterson J, Densley J (2019). We have studied every mass shooting since 1966. Here's what we've learned about the shooters. Los Angeles Times.

[78] Klingner DE, Williams E (2019). Topic: Public Safety. Public Integrity.

[79] Hand C (2016). Gun control and the Second Amendment.

[80] Popovits A (2020). Dominican University of California Political Science & International Studies (Senior Thesis).

[81] Miller SV (2018). What Americans think about gun control: evidence from the General Social Survey, 1972-2016. Soc Sci Quart.

[82] Kellner D, Shapiro H (2018). School shootings, societal violence and gun culture. The Wiley Handbook on Violence in Education: Forms, Factors, and Preventions.

[83] Schildkraut J (2019). Assault weapons, mass shootings, and options for lawmakers. Rockefeller Institute of Government.

[84] Jacobs J, Fuhr Z (2017). The potential and limitations of universal background checking for gun purchasers. Wake Forest J Law Policy.

[85] Braga AA, Brunson RK, Cook PJ, Turchan B, Wade B (2020). Underground gun markets and the flow of illegal guns into the Bronx and Brooklyn: a mixed methods analysis. J Urban Health.

[86] Chai C (2019). Gun control: can we take a shot at it?. AMASS.

[87] Goldberg J (2012). The case for more guns (and more gun control). The Atlantic.

[88] Webster DW, McCourt AD, Crifasi CK, Booty MD, Stuart EA (2020). Evidence concerning the regulation of firearms design, sale, and carrying on fatal mass shootings in the United States. Criminol Public Policy.

[89] Lankford A, Madfis E (2018). Media coverage of mass killers: content, consequences, and solutions. Am Behav Sci.

[90] Kien S, Begay T, Lee A, Stefanidis A (2019). Social media during the school shooting contagion period. Violence Gender.

